# Flavonoid Intake From Cocoa-Based Products and Adiposity Parameters in Adolescents in Spain

**DOI:** 10.3389/fnut.2022.931171

**Published:** 2022-07-06

**Authors:** Emily P. Laveriano-Santos, Camila Arancibia-Riveros, Anna Tresserra-Rimbau, Sara Castro-Barquero, Ana María Ruiz-León, Ramón Estruch, Rosa Casas, Patricia Bodega, Mercedes de Miguel, Amaya de Cos-Gandoy, Jesús Martínez-Gómez, Carla Rodríguez, Gloria Santos-Beneit, Juan M. Fernández-Alvira, Rodrigo Fernández-Jiménez, Rosa M. Lamuela-Raventós

**Affiliations:** ^1^Department of Nutrition, Food Science and Gastronomy, School of Pharmacy and Food Sciences, XIA, Institute of Nutrition and Food Safety (INSA-UB), University of Barcelona, Barcelona, Spain; ^2^Consorcio CIBER, M.P. Fisiopatología de la Obesidad y Nutrición (CIBERObn), Instituto de Salud Carlos III (ISCIII), Madrid, Spain; ^3^Department of Internal Medicine, Hospital Clinic, Institut d'Investigacions Biomèdiques August Pi i Sunyer (IDIBAPS), University of Barcelona, Barcelona, Spain; ^4^Mediterranean Diet Foundation, Barcelona, Spain; ^5^Foundation for Science, Health and Education (SHE), Barcelona, Spain; ^6^Centro Nacional de Investigaciones Cardiovasculares Carlos III (F.S.P.), Madrid, Spain; ^7^The Zena and Michael A. Wiener Cardiovascular Institute, Icahn School of Medicine at Mount Sinai, New York, NY, United States; ^8^CIBER de Enfermedades Cardiovasculares (CIBERCV), Madrid, Spain; ^9^Department of Cardiology, Hospital Universitario Clínico San Carlos, Madrid, Spain

**Keywords:** (poly)phenols, catechin, epicatechin, proanthocyanidins, cardiometabolic, obesity

## Abstract

**Background:**

Cocoa-based products are a good source of flavonoids, which may have beneficial effects on metabolic health.

**Objective:**

The aim of this study is to assess the relationship between flavonoids from cocoa-based products and adiposity parameters in adolescents.

**Methods:**

A cross-sectional study was conducted involving 944 adolescents aged 11–14 years enrolled in the SI! Program for Secondary Schools trial in Spain with available baseline data from food frequency questionnaires and anthropometric measurements [weight, height, waist circumference (WC), and fat mass percentage (% FM) by bioimpedance analysis]. Fat mass index (FMI) and waist-to-height ratio (WHtR) were obtained by dividing fat mass by height and WC by height, respectively. Body mass index (BMI), WC, and FMI for age and gender z-score were calculated. Overweight/obesity was defined as BMI ≥ 85th percentile and excess adiposity as %FM or FMI ≥ 75th percentile. WC ≥ 90th percentile and WHtR with a 0.5 threshold were considered as criteria of abdominal obesity. Multilevel mixed-effect regressions were used to evaluate the association between flavonoids from cocoa-based products and adiposity parameters. Municipalities and schools were considered random effects.

**Results:**

Participants with a higher flavonoid intake from cocoa-based products had lower WC z-score [B = −0.04, 95% CI (−0.07; −0.01), *P-for trend* = 0.045] and WHtR [B = −0.01, 95% CI (−0.02; −0.01), *P- for trend* < 0.001]. They also had lower probability of having abdominal obesity [OR 0.66, 95% CI (0.52; 0.85), *P- for trend* = 0.001]. Inverse associations were observed between flavonoids from cocoa powder and BMI z-score [B = −0.08, 95% CI (−0.12; −0.05), *P* < 0.001], WC z-score [B = −0.06, 95% CI (−0.11; −0.02), *P* = 0.003], WHtR [B = −0.01, 95% CI (−0.01; −0.00), *P* < 0.001], %FM [B = −1.11, 95% CI (−1.48; −0.75), *P* < 0.001], and FMI z-score [B = −0.18, 95% CI (−0.20; −0.17), *P* < 0.001]. Regarding dark chocolate, an inverse association only with WC z-score [B = −0.06, 95% CI (−0.08; −0.05), *P* < 0.001] was found. However, no association was observed between flavonoids from milk chocolate intake and anthropometric parameters.

**Conclusions:**

A higher intake of flavonoids from cocoa-based products was associated with lower adiposity parameters and a lower probability of presenting abdominal obesity.

## Introduction

Obesity, which is characterized by abnormal or excessive body fat accumulation, is a serious public health problem worldwide (1, 2). Excessive adiposity in children and adolescents leads to metabolic disorders such as vascular dysfunction and subclinical indicators of atherosclerosis, increasing the risk of cardiovascular disease and mortality in adulthood (2, 3).

Marked by physiological and emotional changes, adolescence is a critical period for managing obesity. Behavioral modifications, particularly fomenting physical activity and healthy dietary patterns are one of the best strategies used in primary health care settings to reduce obesity among adolescents (2, 4). A diet based on polyphenol-rich foods is of interest because of the antioxidant and anti-inflammatory effect and their influence on physiological and molecular pathways related to body weight maintenance (5–8). The positive impact of cocoa-based products on obesity has been attributed to their content of flavonoids (a large class of phenolic compounds), specifically flavanols (catechins and procyanidins) (9, 10). Systematic reviews and meta-analyses support the beneficial effect of cocoa flavonoids on cardiovascular risk factors since they are reported to favorably improve blood pressure, lipid profile, inflammation, and adiposity parameters (11–13). However, as most of the research in this field has been performed in adults, there is a need for studies on adolescents to establish dietary recommendations for the consumption of cocoa-based products in this target population, always within the framework of a healthy lifestyle. Therefore, this study aimed to investigate the association between flavonoid intake from cocoa-based products and adiposity parameters in a large sample of adolescents in Spain.

## Materials and Methods

### Study Population

The SI! (*Salud Integral-Comprehensive Health*) Program for Secondary Schools trial (NCT03504059) is a cluster-randomized controlled intervention trial conducted in adolescents from 24 secondary schools in Spain and conducted from 2017 to 2021. The main objective of this trial was to evaluate the effectiveness of an educational intervention to promote cardiovascular health at schools. A detailed description of the original study design and recruitment procedures has been previously published (14). Parents or caregivers provided assent and written informed consent before entering the study.

**Figure 1:**
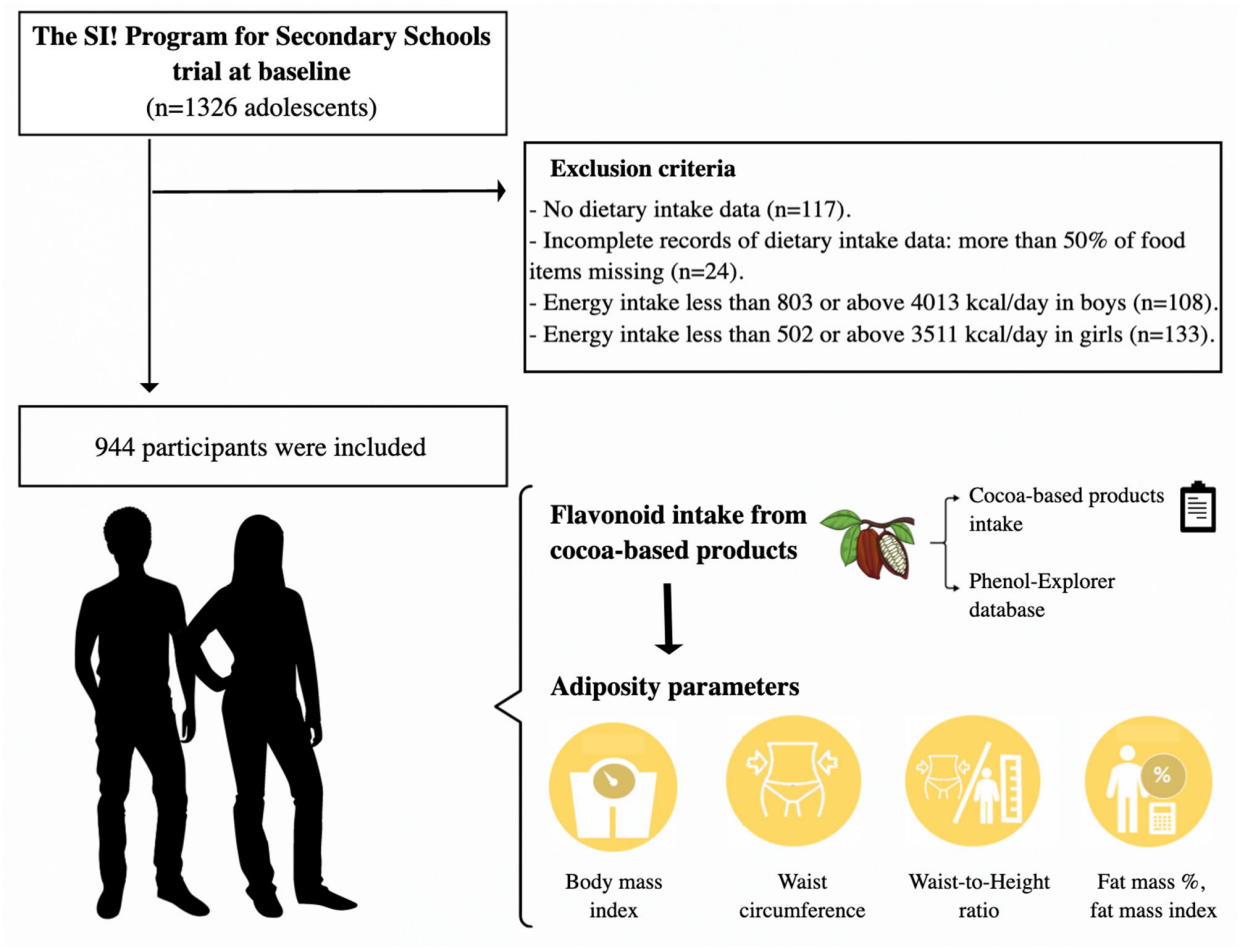
Study design of the study.

The present cross-sectional study derived from the SI! Program for Secondary Schools trial was carried out using baseline data (2017) collected from 944 participants with available information on food consumption frequency, and whose total energy intake ranged from 803 to 4,013 kcal/day in boys and 502 to 3,511 kcal/day in girls (15) (Figure 1).

### Assessment of Flavonoids From Cocoa-Based Products

Dietary intake was assessed by a validated semi-quantitative food frequency questionnaire (15, 16). Cocoa-based product intake was expressed in grams and included cocoa powder (25% of pure cocoa), dark (more than 70% pure cocoa), and milk chocolate (about 30% pure cocoa). Cookies, pastries, and beverages made of cocoa-based products were not considered due to the lack of information on their content of flavonoids. Flavonoids from cocoa-based products were estimated using the Phenol-Explorer database (http://www.phenol-explorer.eu), which included flavanols, such as catechin, epicatechin, cinnamtannins, and proanthocyanidins (dimers, trimers, 4–6 mers, 7–10 mers, polymers, and monomers), and flavonol-like quercetin (17, 18). In brief, flavonoids from cocoa-based products were estimated (mg/100 g fresh food weight) for each food and then multiplied by intake of the respective foods (g/day). Total flavonoids from cocoa-based products were estimated as the sum of intakes of the individual flavonoids (catechin, epicatechin, cinnamtannins, proanthocyanidins, and quercetin). Energy-adjusted flavonoid intake was calculated by the residual method established by Willet et al. (19).

### Assessment of Adiposity Parameters

All participants were evaluated by trained staff, who performed the anthropometric measurements of weight, height, and waist circumference (WC) according to standard procedures (14). Weight was obtained to the nearest 0.1 kg using a digital scale (OMRON BF511) and height to the nearest 0.1 cm with a portable SECA 213 stadiometer. WC was measured to the nearest 0.1 cm. To minimize measurement errors, WC was measured three times and a mean value was calculated. The percentage of fat mass (%FM) was estimated by bioelectrical impedance using a tetrapolar OMRON BF511 and fat mass weight was calculated as the product of fat percentage and body weight.

Body mass index (BMI) was calculated as body weight in kilograms divided by height squared in meters (kg/m^2^). The fat mass index (FMI) and waist-to-height ratio (WHtR) were obtained by dividing fat weight (kg) by height squared (m^2^) and WC (cm) by height (cm), respectively. Age- and gender-specific BMI, WC z-scores, and FMI z-scores (standard deviation score) were calculated according to the Center for Disease Control growth references and the National Health and Nutrition Examination Survey data (20–23).

Overweight was defined as BMI at or above the 85th percentile to less than the 95th percentile, and obesity as equal to or greater than the BMI 95th percentile (21, 22). Participants with a BMI percentile equal to or above the 85th percentile were classified as overweight/obese (21, 22). Abdominal obesity was defined by WC at or above the 90th percentile and/or WHtR equal to or above the 0.5 threshold (20, 24, 25). Finally, participants with a %FM and/or FMI greater than or equal to the age- and gender-specific 75th percentile were classified as excess adiposity, according to published reference data for %FM and FMI (23, 26).

### Assessment of Covariates

Intake of energy, foods, and nutrients was determined from the semi-quantitative food frequency questionnaire that was previously described, with the use of values from Spanish food composition tables (27, 28).

Information on physical activity was obtained with the use of accelerometers and a standardized questionnaire. Physical activity was measured using accelerometers (Actigraph wGT3X-BT, ActiGraph, Pensacola, USA) worn on the non-dominant wrist for 7 days, except during water-based activities (14). Moderate-to-vigorous physical activity was estimated according to the cut points of Chandler et al. and is presented as the average minutes of moderate-to-vigorous physical activity per day (29). In participants with missing accelerometer data (*n* = 48), the information from the QAPACE survey (*Quantification de L'Activité Physique en Altitude chez les Enfants*) was used to estimate moderate-to-vigorous physical activity according to the frequency and duration of recreational physical activity and competitive sports performed inside or outside schools, on school days, and at weekends (14, 30). A conversion factor was used to calculate moderate-to-vigorous physical activity in terms of minutes per day. For the analysis, physical activity was categorized as below or equal to/above 60 min/day of moderate-to-vigorous physical activity based on physical activity recommendations for adolescents by the World Health Organization (31).

Information about parental education was obtained from a general questionnaire answered by the parents (14). A high level of parental education corresponded to university studies according to the International Standard Classification of Education (32). Puberty development was categorized according to the Tanner maturation stages using pictograms (33).

### Statistical Analysis

A descriptive analysis of the population was carried out using mean (SD) and frequency distribution. Participants were categorized into quintiles of energy-adjusted flavonoids from cocoa-based products (Q1: <12.1, Q2: 12.1–32.0, Q3: 32.1–53.2, Q4: 53.3–83.8, and Q5 >83.8 mg/day). One-way analysis of variance, including Bonferroni *post-hoc* test, and chi-square analysis were performed to assess the differences in means and frequencies across quintiles of flavonoids from cocoa-based products, respectively.

Multilevel mixed-effects linear regression models with robust error variance were used to evaluate the association between quintiles of flavonoids from cocoa-based products with the anthropometric measurements (BMI *z*-score, WC *z*-score, WHtR, %FM, and FMI *z*-score) as continuous variables. Multilevel generalized logistic regression was performed to study the association between quintiles of flavonoids from cocoa-based products and adiposity parameters (BMI ≥ 80th to <95th percentile, BMI ≥ 95th percentile, WC ≥ 90th percentile, and WHtR ≥ 0.5). The fixed effects were gender (girls/boys), age (continuous, year), Tanner maturation stage (from I to V), physical activity (≥60 min/ <60 min moderate-to-vigorous physical activity), parental education (university studies/lower than university studies), intake of energy (continuous, Kcal/day), sweetened products like breakfast cereals (continuous, g/day), pastries (continuous, g/day), sugar-sweetened beverages (continuous, g/day), meat, and processed meat (continuous, g/day), and other polyphenol-rich food intakes like fruits (continuous, g/day), vegetables (continuous, g/day), legumes (continuous, g/day), nuts (continuous, g/day), and extra olive oil (continuous, g/day). Municipalities (Barcelona/Madrid) and schools were included as random effects. Gender interaction was considered to evaluate potential effect modification in the association between flavonoids from cocoa-based products and adiposity parameters. Orthogonal polynomial contrast was used to determine linear trends.

In addition, a multilevel mixed-effects linear regression analysis was conducted to explore associations between flavonoids of each cocoa-based product (cocoa powder, dark chocolate, and milk chocolate) and adiposity parameters, all of them as continuous variables. For this analysis, data from participants who reported daily intake of at least one cocoa-based product were considered (700 participants reported cocoa powder intake, 294 reported dark chocolate intake, and 644 reported milk chocolate intake). The model included the same fixed and random effects variables as described earlier. Moreover, Pearson correlation coefficients were used to explore the relationship between individual flavonoids from cocoa-based products and adiposity parameters. Finally, the false discovery rate (FDR) by the Benjamini-Hochberg procedure was applied to adjust *p*-values for multiple correlations (34). Before these analyses, values of flavonoids were normalized and scaled in 1-SD with the inverse normal transformation (35).

All statistical analyses were conducted using Stata statistical software package version 16.0 (StataCorp., College Station, TX, USA) and R 4.1.1 (R Foundation for Statistical Computing, Vienna, Austria). Statistical tests were two-sided and statistical significance was set as 0.05.

## Results

### General Characteristics of the Study Participants

The characteristics of the cohort stratified by quintiles of flavonoid intake from cocoa-based products are shown in Table 1. Based on BMI z-score, 18% of adolescents presented overweight and 9% obesity. Regarding the abdominal obesity parameters, 16% of participants had a WC greater than the 90th percentile, and 23% had a high WHtR (≥0.5 threshold). Finally, regarding the excess of adiposity, 8 and 16% of adolescents had %FM and FMI equal to or greater than the 75th percentile, respectively. Compared to the lowest quintile, participants in the highest quintile tended to have slightly lower BMI, WC, and WC *z*-score, although the differences were not significant in the univariate analysis.

**Table 1:** Characteristics of participants according to quintiles of flavonoid intake from cocoa-based products (mg/day).

	**Overall (*n* = 944)**	**Q1 (<12.1)**	**Q2 (12.1–32.0)**	**Q3 (32.1–53.2)**	**Q4 (53.3–83.8)**	**Q5 (>83.8)**	***P-for trend***
		**(*n* = 189)**	**(*n* = 189)**	**(*n* = 189)**	**(*n* = 189)**	**(*n* = 188)**	
Girls, *n* (%)	455 (48)	76 (40)	101 (53)	89 (47)	90 (48)	99 (53)	0.098
Age, years	12.0 (0.4)	12.0 (0.4)	12.0 (0.4)	12.0 (0.4)	12.0 (0.4)	12.0 (0.4)	0.740
**Anthropometric measurements**							
BMI, kg/m^2^	20.2 (3.7)	20.2 (3.6)	20.4 (3.9)	20.5 (4.1)	20.4 (3.7)	19.7 (3.3)	0.286
WC, cm	71.9 (10.1)	72.2 (10.1)	71.7 (10.1)	72.7 (11.6)	72.5 (9.9)	70.5 (8.7)	0.232
WHtR	0.4 (0.1)	0.5 (0.1)	0.4 (0.1)	0.5 (0.1)	0.5 (0.1)	0.5 (0.1)	0.082
%FM	23.3 (8.3)	22.7 (8.2)	23.5 (8.1)	23.7 (9.0)	23.9 (8.2)	22.7 (8.0)	0.980
FMI, kg/m^2^	5.0 (2.7)	4.9 (2.6)	5.1 (2.8)	5.2 (2.9)	5.1 (2.6)	4.7 (2.4)	0.656
**Adiposity parameters**, ***n*** **(%)**							
BMI ≥ 85th to <95th percentile	172 (18)	39 (21)	33 (18)	32 (17)	41 (22)	27 (14)	0.334
BMI ≥ 95th percentile	89 (9)	14 (7)	18 (10)	24 (13)	19 (10)	14 (7)	0.943
WC ≥ 90th percentile	153 (16)	34 (18)	25 (13)	36 (19)	33 (17)	25 (13)	0.546
WHtR ≥ 0.5	213 (23)	48 (25)	39 (21)	44 (23)	50 (26)	32 (17)	0.258
%FM ≥ 75th percentile	79 (8)	14 (7)	14 (7)	19 (10)	17 (9)	15 (8)	0.646
FMI ≥ 75th percentile	146 (16)	28 (15)	26 (14)	37 (20)	31 (16)	24 (13)	0.888
**Physical activity**, ***n*** **(%)**							
≥60 min/day MVPA	310 (33)	71 (38)	64 (34)	57 (30)	61 (32)	57 (30)	0.137
**Parental education**, ***n*** **(%)**							
University level	240 (26)	58 (31)	44 (24)	49 (27)	46 (25)	43 (24)	0.165
**Municipality**, ***n*** **(%)**							**0.021**
Barcelona	644 (68)	138 (73)	141 (75)	121 (64)	119 (63)	125 (66)	
Madrid	300 (32)	51 (27)	48 (25)	68 (36)	70 (37)	63 (34)	

*Data are expressed as mean (SD) or frequency (percentage)*.

*Q, quintiles of flavonoids from cocoa-based products; n, number; SD, standard deviation; BMI, body mass index; WC, waist circumference; WHtR, waist-to-height ratio; %FM, body fat percentage; FMI, fat mass index; MVPA, moderate-to-vigorous physical activity*.

*Statistical analyses were conducted using one-way ANOVA for continuous variables and the chi-square test for categorical variables. P-for trend were obtained using orthogonal contrasts test. P < 0.05 are considered statistically significant*.

*Significant differences are bolded*.

The mean cocoa-based product intake was 7.4 (7.6) g/d, equivalent to one tablespoon of cocoa powder or one square piece of a chocolate bar. More than 90% of the participants reported daily intake of at least one cocoa-based product, from them, 75% (*N* = 700) reported intake of cocoa powder, 31% (*N* = 294) dark chocolate, and 68% (*N* = 644) milk chocolate. The mean flavonoid intake from cocoa-based products was 57.4 (74.5) mg/day, where 26.6 (35.3) mg/day were from cocoa powder, 24.0 (62.9 mg/day) from dark chocolate, and 6.7 (11.3) mg/day from milk chocolate (Data not shown). Participants with a higher intake of flavonoids from cocoa-based products tended to consume lower fish, meat, processed meat, refined grains, (poly)phenol-rich foods (legumes, vegetables, fruits, and nuts), and sugar-sweetened beverages (Table 2). In addition, compared to the lowest quintile, participants in the highest quintile of flavonoids from cocoa-based products had a lower energy intake and macro and micronutrients (Table 3), except for vitamin A. A higher intake of total dietary flavonoids was observed in the highest quintile, but lower values of phenolic acids, lignans, and other (poly)phenol intake were observed in the same group (Table 3).

**Table 2:** Dietary food intake of participants according to quintiles of flavonoids from cocoa-based products (mg/day).

	**Overall (*n* = 944)**	**Q1 (<12.1)**	**Q2 (12.1–32.0)**	**Q3 (32.1–53.2)**	**Q4 (53.3–83.8)**	**Q5 (>83.8)**	***P-for trend***
		**(*n* = 189)**	**(*n* = 189)**	**(*n* = 189)**	**(*n* = 189)**	**(*n* = 188)**	
Fish products, g/day	86.6 (55)	103.3 (67.2)^a^	89.2 (52.6)^a,b^	81.6 (47.0)^b^	76.1 (49.6)^b^	82.9 (53.1)^b^	**<0.001**
Meat, g/day	172.6 (90.4)	207.4 (107.9)^a^	161.2 (82.8)^b^	172.5 (87.5)^b^	155.8 (75.2)^b^	166.0 (86.6)^b^	**<0.001**
Processed meat, g/day	7.0 (6.9)	8.7 (8.5)^a^	7.1 (7.1)^a,b,c^	6.6 (6.3)^b,c^	6.7 (6.3)^a,b,c^	6.1 (5.4)^c^	**0.001**
Dairy products, g/day	406.6 (253.9)	448.4 (275.8)^a^	404.9 (254.3)^a,b^	382.4 (231.5)^a,b^	357.7 (193.6)^b^	439.9 (293.3)^a^	0.269
Refined grains, g/day	113.2 (69.4)	139.8 (85.8)^a^	115.6 (70.1)^b^	107.2 (59.1)^b^	105.7 (62.9)^b^	97.6 (58.7)^b^	**<0.001**
Wholegrains, g/day	18.5 (32.2)	19.2 (33.6)	18.9 (32.0)	15.1 (25.9)	19.7 (34.1)	19.7 (34.8)	0.814
Breakfast cereals, g/day	14.6 (19.7)	17.8 (29.1)^a^	15.3 (18.8)^a^	13.4 (15.2)^a^	14.6 (16.9)^a^	12.1 (14.7)^a^	**0.008**
Legumes, g/day	60.6 (44.2)	72.0 (64.7)^a^	59.6 (37.8)^a,b^	58.4 (36.2)^b^	57.2 (37.3)^b^	55.6 (36.4)^b^	**0.001**
Vegetables, g/day	205.3 (150.6)	246.4 (199.8)^a^	200.9 (137.9)^b^	194.9 (134.7)^b^	190.4 (129.7)^b^	193.5 (133.1)^b^	**0.001**
Fruits, g/day	334.7 (250.7)	430.6 (290.0)^a^	338.6 (216.2)^b^	310.3 (243.1)^b^	292.4 (208.5)^b^	301.1 (263.3)^b^	**<0.001**
Nuts, g/day	11.3 (14.5)	16.1 (19.7)^a^	10.8 (13.7)^b^	9.4 (10.6)^b^	9.7 (11.7)^b^	10.6 (14.1)^b^	**<0.001**
Olive oil, g/day	16.6 (14.5)	17.6 (13.6)	17.2 (17.2)	16.9 (13.6)	15.3 (12.6)	16.1 (15.0)	0.141
Cocoa-based products, g/day	7.4 (7.6)	2.6 (2.5)^a^	3.9 (3.5)^a^	5.7 (4.1)^b^	8.0 (5.3)^c^	16.6 (10.3)^d^	**<0.001**
Sugar-sweetened beverages, g/day	54.8 (95.1)	73.2 (123.2)^a^	52.4 (96.5)^a,b^	50.6 (94.5)^a,b^	55.3 (81.3)^a,b^	42.3 (69.4)^b^	**0.007**
Pastry products, g/day	69.1 (52.5)	74.2 (54.6)	67.2 (53.9)	68.4 (53.5)	66.5 (46.6)	73.0 (53.2)	0.561

*Data are expressed as mean (SD)*.

*Q, quintiles of flavonoids from cocoa-based products; n, number; SD, standard deviation*.

*Statistical analyses were conducted using one-way ANOVA for continuous variables and the chi-square test for categorical variables*.

a,b,c,d *Data sharing the different letters are statistically different after Bonferroni post-hoc test. P-for trend were obtained using orthogonal contrasts test. P < 0.05 are considered statistically significant*.

*Significant differences are bolded*.

**Table 3:** Nutrients and (poly)phenols intake of participants according to quintiles of flavonoids from cocoa-based products (mg/day).

	**Overall (*n* = 944)**	**Q1 (<12.1)**	**Q2 (12.1–32.0)**	**Q3 (32.1–53.2)**	**Q4 (53.3–83.8)**	**Q5 (>83.8)**	***P-for trend***
		**(*n* = 189)**	**(*n* = 189)**	**(*n* = 189)**	**(*n* = 189)**	**(*n* = 188)**	
**Nutrients intake**							
Energy, Kcal/day	2,539.2 (601.8)	3,013.4 (435.9)^a^	2,510.9 (490.9)^b^	2,402.6 (562.7)^b,c^	2,307.3 (614.7)^c^	2,461.3 (622.6)^b,c^	**<0.001**
Carbohydrates, g/day	256.5 (72.2)	303.2 (65.8)^a^	255.8 (65.7)^b^	240.6 (64.3)^b^	236.3 (71.3)^b^	246.7 (73.3)^b^	**<0.001**
Fiber, g/day	29.4 (10.7)	35.5 (11.4)^a^	29.1 (8.5)^b^	27.6 (9.9)^b^	27.2 (10.3)^b^	27.6 (10.7)^b^	**<0.001**
Proteins, g/day	120.9 (33.3)	144.2 (30.1)^a^	118.9 (27.8)^b^	115.9 (30.8)^b,c^	108.3 (30.3)^c^	117.5 (35.5)^b^	**<0.001**
SFA, g/day	36.8 (11.5)	44.2 (11.3)^a^	35.8 (9.5)^b^	34.6 (10.6)^b,c^	32.7 (10.8)^c^	36.8 (11.7)^b^	**<0.001**
MUFA, g/day	48.5 (16.2)	57.0 (14.2)^a^	48.1 (15.9)^b^	46.4 (15.3)^b^	43.8 (15.8)^b^	47.2 (16.5)^b^	**<0.001**
PUFA, g/day	19.7 (6.8)	23.7 (6.4)^a^	19.4 (5.7)^b^	18.7 (6.2)^b^	18.2 (7.3)^b^	18.5 (6.9)^b^	**<0.001**
Calcium, mg/day	1,012.9 (391.0)	1,198.1 (403.5)^a^	1,001.6 (354.7)^b,c^	959.7 (362.2)^b,c^	896.9 (321.2)^b^	1,008.1 (440.3)^c^	**<0.001**
Vitamin A, μg/day	1,476.0 (1,465.2)	1,849.9 (1,719.5)^a^	1,542.8 (1,759.5)^a,b^	1,342.6 (1,107.7)^b^	1,177.2 (754.9)^b^	1,467.6 (1,637.2)^a,b^	**0.001**
Vitamin D, μg/day	5.1 (2.6)	6.1 (3.0)^a^	5.2 (2.4)^b^	4.8 (2.4)^b^	4.6 (2.5)^b^	4.7 (2.4)^b^	**<0.001**
**(Polyp)phenols intake**							
Flavonoids, mg/day	530.1 (331.3)	482.8 (314.0)^a^	440.1 (283.9)^a^	460.6 (263.3)^a^	490.4 (241.1)^a^	777.8 (406.1)^b^	**<0.001**
Phenolic acids, mg/day	97.8 (64.5)	117.2 (70.8)^a^	103.5 (74.4)^b^	89.6 (53.8)^b^	89.4 (60.4)^b^	89.3 (56.5)^b^	**<0.001**
Stilbenes, mg/day	0.2 (0.3)	0.2 (0.3)	0.2 (0.3)	0.2 (0.3)	0.2 (0.3)	0.2 (0.4)	0.677
Lignans, mg/day	3.8 (5.1)	5.4 (6.1)^a^	3.2 (3.7)^b^	3.5 (5.3)^b^	3.7 (5.7)^b^	3.4 (4.1)^b^	**0.002**
Other, mg/day	51.3 (34.4)	59.5 (44.0)^a^	55.1 (34.7)^a,b^	46.5 (29.7)^b^	48.9 (30.8)^b^	46.5 (28.6)^b^	**<0.001**

*Data are expressed as mean (SD)*.

*Q, quintiles of flavonoids from cocoa-based products; n, number; SD, standard deviation; SFA, saturated fatty acids; MUFA, monounsaturated fatty acids; PUFA, polyunsaturated fatty acids. Statistical analyses were conducted using one-way ANOVA for continuous variables and the chi-square test for categorical variables*.

a,b,c *Data sharing the different letters are statistically different after Bonferroni post-hoc test. P-for trend were obtained using orthogonal contrasts test. P < 0.05 are considered statistically significant*.

*Significant differences are bolded*.

### Association of Dietary Flavonoids From Cocoa-Based Products With Adiposity Parameters

The results from the multivariate-adjusted linear regression analyses showed that a higher intake of flavonoids from cocoa-based products was associated with lower values of BMI z-score (*P-for trend* < 0.001); however, no significant difference was observed between the highest and lowest quintiles. Moreover, participants with highest intake of flavonoids from cocoa-based products had lower values of WC z-score [B = −0.04, 95% CI (−0.07; −0.01), *P-for trend* = 0.045] and WHtR [B = −0.01, 95% CI (−0.02; −0.01), *P-for trend* < 0.001] (Table 4). However, quartiles 2 and 3 had higher values of BMI *z*-score, %FM, and FMI *z*-score compared to quartile 1. No interaction with gender was found in the regression analysis.

**Table 4:** Association between flavonoid intake from cocoa-based products (mg/day) and anthropometric measurements.

**Anthropometric variables**	**Q1 (<12.1)**	**Q2 (12.1–32.0)**	**Q3 (32.1–53.2)**	**Q4 (53.3–83.8)**	**Q5 (>83.8)**	***P-for trend***
		**(ß, 95% CI)**	**(ß, 95% CI)**	**(ß, 95% CI)**	**(ß, 95% CI)**	
BMI *z*-score	Reference	0.25 (0.10; 0.40)	0.29 (0.17; 0.41)	0.06 (−0.04; 0.15)	−0.07 (−0.25; 0.10)	**<0.001**
WC *z*-score	Reference	0.09 (−0.01; 0.19)	0.23 (0.17; 0.29)	0.06 (−0.13; 0.24)	−0.04 (−0.07; −0.01)	**0.045**
WHtR	Reference	−0.00 (−0.01; 0.01)	0.01 (0.01; 0.01)	−0.00 (−0.00; −0.00)	−0.01 (−0.02; −0.01)	**<0.001**
%FM	Reference	0.72 (0.51; 0.94)	2.17 (1.95; 2.38)	0.09 (−0.14; 0.32)	−1.15 (−3.36; 1.05)	0.160
FMI *z*-score	Reference	0.41 (0.18; 0.64)	0.52 (0.39; 0.66)	0.21 (0.02; 0.40)	0.02 (−0.53; 0.56)	0.242

*Q, quintiles of flavonoids from cocoa-based products (mg/day); ß, (beta) regression coefficient; CI, confidence interval; BMI, body mass index; WC, waist circumference; WHtR, waist-to-height ratio; %FM, percentage of fat mass; FMI, fat mass index*.

*Statistical analyses were conducted using multilevel mixed-effect linear regression analysis. The fixed effects were gender, age, Tanner maturation stage, physical activity, parental education, intake of energy, breakfast cereals, pastries, sugar-sweetened beverages, meat, processed meat, fruits, vegetables, legumes, nuts, and extra olive oil. Municipalities and schools were included as random effects. P-for trend were obtained using orthogonal contrasts test across quintiles. P < 0.05 are considered statistically significant*.

*Significant differences are bolded*.

Table 5 shows the association of flavonoids from each cocoa-based product and anthropometric parameters. Inverse associations were observed between flavonoids from cocoa powder and BMI *z*-score [B = −0.08, 95% CI (−0.12; −0.05), *P* < 0.001], WC *z*-score [B = −0.06, 95% CI [−0.11; −0.02], *P* = 0.003], WHtR [B = −0.01, 95% CI (−0.01; −0.00), *P* < 0.001], %FM [B = −1.11, 95% CI (−1.48; −0.75), *P* < 0.001], and FMI *z*-score [B = −0.18, 95% CI (−0.20; −0.17), *P* < 0.001]. Regarding dark chocolate, an inverse association only with WC *z*-score [B = −0.06, 95% CI (−0.08; −0.05), *P* < 0.001] was found. However, no association was observed between flavonoids from milk chocolate intake and anthropometric parameters.

**Table 5:** Association between flavonoids from cocoa powder, dark chocolate, and milk chocolate (mg/day) and anthropometric measurements.

**Anthropometric measurements**	**Cocoa powder**	***P***	**Dark chocolate**	***P***	**Milk chocolate**	***P***
	***N* = 700**		***N* = 294**		***N* = 644**	
	**(ß, 95% CI)**		**(ß, 95% CI)**		**(ß, 95% CI)**	
BMI *z*-score	−0.08 (−0.12; −0.05)	**<0.001**	−0.11 (−0.23; 0.12)	0.076	−0.02 (−0.11; 0.06)	0.593
WC *z*-score	−0.06 (−0.11; −0.02)	**0.003**	−0.06 (−0.08; −0.05)	**<0.001**	−0.01 (−0.02; 0.01)	0.403
WHtR	−0.01 (−0.01; −0.00)	**<0.001**	−0.003 (−0.01; 0.00)	0.110	−0.001 (−0.00; 0.00)	0.646
%FM	−1.11 (−1.48; −0.75)	**<0.001**	−0.42 (−1.63; 0.79)	0.494	0.112 (−0.49; 0.72)	0.718
FMI *z*-score	−0.18 (−0.20; −0.17)	**<0.001**	−0.16 (−0.36; 0.06)	0.147	−0.01 (−0.13; 0.11)	0.822

*N, number of participants who reported cocoa powder, dark chocolate, or milk chocolate intake; ß, regression coefficient; CI, confidence interval; BMI, body mass index; WC, waist circumference; WHtR, waist-to-height ratio; %FM, body fat percentage; FMI, fat mass index*.

*Statistical analyses were conducted using multilevel mixed-effect linear regression analysis. The fixed effects were gender, age, Tanner maturation stage, physical activity, parental education, intake of energy, breakfast cereals, pastries, sugar-sweetened beverages, meat, processed meat, fruits, vegetables, legumes, nuts, and extra olive oil. Municipalities and schools were included as random effects. Data from flavonoids were normalized with the inverse normal distribution before this analysis. P < 0.05 are statistically significant*.

*Significant differences are bolded*.

**Figure 2:**
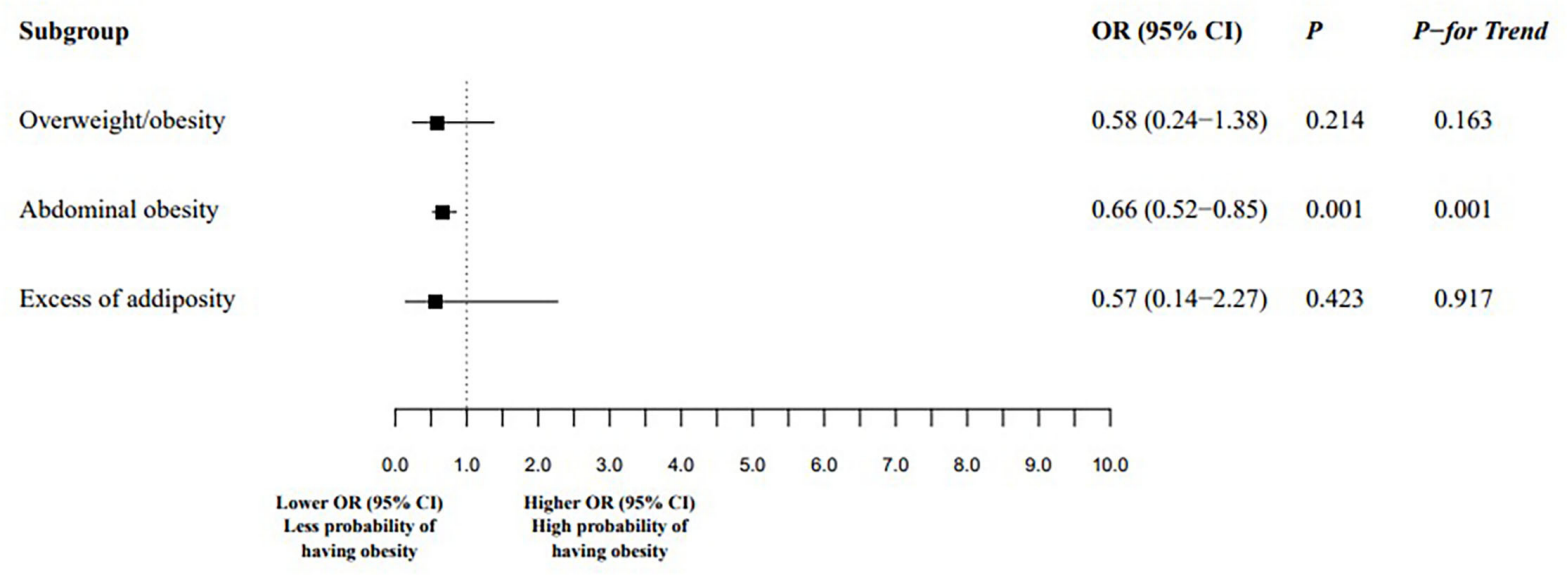
Association between the highest and the lowest quintiles of flavonoids from cocoa-based products (mg/day) intake and obesity. Overweight/obesity was defined by body mass index percentile equal to or above the age- and gender-specific 85th percentile. Abdominal obesity was defined by WC at or above the 90th percentile and/or WHtR equal to or above the 0.5 threshold. Excess of adiposity was defined by %FM and or FMI greater than or equal to the age- and gender-specific 75th percentile. Q, quintiles of flavonoids from cocoa-based products; OR, odds ratio; CI, confidence interval. Statistical analyses were conducted using multilevel mixed-effect logistic regression model. The fixed effects were gender, age, Tanner maturation stage, physical activity, parental education, intake of energy, breakfast cereals, pastries, sugar-sweetened beverages, meat, processed meat, fruits, vegetables, legumes, nuts, and extra olive oil. Municipalities and schools were included as random effects. *P*-values between Q5 vs. Q1 and *P*- *for trend* were obtained using orthogonal contrasts test. *P* < 0.05 are considered statistically significant.

Multivariate-adjusted logistic regression analyses revealed a tendency of having less probability of having overweight (BMI at or above the 85th percentile to less than the 95th percentile) and high WHtR (≥0.5 thresholds) in participants with higher flavonoid intake from cocoa-based products (Table 6). In addition, participants in the highest quintile had less probability of having abdominal obesity [OR 0.66, 95% CI (0.52; 0.85), *P- for trend* = 0.001] compared to the lowest quintile (Figure 2). However, participants in quintiles 4 and 3 had a higher probability of obesity (BMI at or above 95th percentile) compared to quintile 1.

**Table 6:** Association between flavonoids from cocoa-based products (mg/day) and adiposity parameters.

**Adiposity parameters**	**Q1 (<12.1)**	**Q2 (12.1–32.0)**	**Q3 (32.1–53.2)**	**Q4 (53.3–83.8)**	**Q5 (>83.8)**	***P-for trend***
		**(OR, 95% CI)**	**(OR, 95% CI)**	**(OR, 95% CI)**	**(OR, 95% CI)**	
BMI ≥ 85th to <95th percentile	1	1.17 (1.11; 1.22)	0.94 (0.86; 1.03)	0.93 (0.51; 1.71)	0.52 (0.20; 1.35)	**0.023**
BMI ≥ 95th percentile	1	1.11 (0.76; 1.61)	2.86 (2.40; 3.41)	1.41 (1.22; 1.62)	0.96 (0.63; 1.45)	0.908
WC ≥ 90th percentile	1	0.52 (0.34; 0.80)	1.48 (1.16; 1.87)	0.83 (0.61; 1.14)	0.79 (0.68; 0.93)	0.520
WHtR ≥ 0.5	1	0.97 (0.54; 1.74)	1.14 (0.91; 1.42)	0.96 (0.68; 1.36)	0.60 (0.33; 1.09)	**<0.001**
%FM ≥ 85th percentile	1	0.79 (0.69; 0.90)	1.95 (1.45; 2.62)	1.02 (0.53; 1.98)	1.02 (0.53; 1.98)	0.736
FMI ≥ 75th percentile	1	0.75 (0.49; 1.14)	1.59 (1.42; 1.77)	0.92 (0.74; 1.16)	0.56 (0.14; 2.22)	0.859

*Q, quintiles of flavonoids from cocoa-based products; OR, odds ratio; CI, confidence interval; BMI, body mass index; WC, waist circumference; WHtR, waist-to-height ratio; %FM, body fat percentage; FMI, fat mass index*.

*Statistical analyses were conducted using multilevel mixed-effect logistic regression model. The fixed effects were gender, age, Tanner maturation stage, physical activity, parental education, intake of energy, breakfast cereals, pastries, sugar-sweetened beverages, meat, processed meat, fruits, vegetables, legumes, nuts, and extra olive oil. Municipalities and schools were included as random effects. P-for trend were obtained using orthogonal contrasts test. P < 0.05 are considered statistically significant*.

*Significant differences are bolded*.

Finally, in the correlation analysis between individual flavonoids from cocoa-based products and adiposity parameters, weak inverse correlations between WHtR and catechins (R = −0.08, FDR value = 0.027), epicatechins (R = −0.09, FDR value = 0.014), and proanthocyanidins (R = −0.08, FDR value = 0.021) were observed (Data not shown).

## Discussion

In the present study, a higher intake of flavonoids from cocoa-based products was inversely associated with individual adiposity parameters and abdominal obesity in adolescents. To our knowledge, this is one of the first studies to explore these associations in this target population.

### Cocoa Flavonoid Intake in Adolescents

Cocoa-based products are an important dietary source of flavonoids. In our study, adolescents consumed a mean of 57.4 mg/day of flavonoids from cocoa-based products, representing 11% of the total dietary flavonoid intake (mean 530.1 mg/day). A lower intake of flavonoids from cocoa-based products was reported by Bawaked et al. in Spanish children aged 6 to 11 years, who consumed 10.9 mg/day of flavonoids from cocoa powder and chocolate, which provided 23.5% of the total flavonoid intake (mean 70.7 mg/day) (36). In the Healthy Lifestyle in Europe by Nutrition in Adolescence study, chocolate products were once the major source of dietary (poly)phenols and flavonoids in European adolescents (37).

### Flavonoids From Cocoa-Based Products and Adiposity Parameters

The inverse association of flavonoids from cocoa-based products with adiposity parameters is in accordance with previous studies, although most of them were conducted in adults. The fact that the results remained consistent when using different adiposity parameters (BMI *z*-score, WC *z*-score, WHtR, %FM, and FMI *z*-score) further strengthens the study findings. In addition, in our cross-sectional, multivariate-adjusted model, clinical relevance was observed between extremes of quintiles of flavonoids from cocoa-based products (Q5 vs. Q1) and less probability of having abdominal obesity. These results were independent of physical activity, puberty development, parental education, intake of energy, sweetened products, meat, and processed meat, as well as other (poly)phenol-rich foods intake such as fruits, vegetables, legumes, nuts, and extra olive oil. Fruits and vegetables represent the main food source of flavonoids in the diet of adolescents and their consumption could influence the association of flavonoids from cocoa-based products with adiposity parameters (36, 38–40). According to the results of a cross-sectional study of European adolescents, consumption of energy-dense foods is associated with a higher probability of obesity (41). In our study, a tendency to consume less energy-dense and sugary foods was observed in participants with a higher intake of flavonoids from cocoa-based products.

In agreement with our findings, Cuenca-García et al. recently reported that higher chocolate consumption was associated with lower BMI, body fat, and WC in European adolescents (40). Similar results were obtained in a large cross-sectional analysis in non-diabetic US adults, where the BMI and WC of individuals who reported chocolate intake were lower by 0.92 Kg/m^2^ and 2.07 cm, respectively, compared to the non-reporters, a difference that could be attributed to the intake of cocoa flavonoids (42). However, the authors did not define the type of chocolate being consumed (for example, dark or milk), and its flavonoid content was not calculated. In our study, inverse associations between flavonoids from cocoa powder and BMI *z*-score, WC *z*-score, WHtR, %FM, and FMI *z*-score were observed, but WC *z*-score was inversely associated only with dark chocolate. No association between anthropometric parameters and milk chocolate was found. These results could be attributed to the highest concentration of flavonoids in cocoa powder compared to dark or milk chocolate (17, 18, 43). In addition to this, it could be also explained by the fact that cocoa powder was consumed by most participants (75%), with fewer consuming milk (68%) and dark chocolates (31%).

Evidence between cocoa flavonoid consumption and adiposity in adults is conflicting. The results of a meta-analysis based on randomized clinical trials suggested that the consumption of at least 30 g/day of cocoa/dark chocolate for 4–8 weeks decreases BMI in adults (12). Similarly, weight reduction in overweight/obese adults (BMI ≥ 25 kg/m^2^) was related to the consumption of flavanol-containing products such as tea, cocoa, and apple in a subgroup meta-analysis (13). In contrast, a meta-analysis of short-term trials did not find significant associations between flavonoid intake from cocoa-based products and BMI in adults, although this could have been due to the short-term nature of the studies (44). Longer-term randomized controlled clinical trials are needed to examine the magnitude of the effect of flavonoids from cocoa-based products on adiposity parameters. Instead, the limitations of BMI as an adiposity indicator are well-known, because it does not provide information on adiposity distribution, and therefore additional anthropometric measurements are required, such as WC, WHtR, %FM, and FMI (24, 45).

Cocoa-based products also contain other bioactive compounds like theobromine, a methylxanthine highly associated with body weight, lipid, and glucose metabolism (46). In our study, theobromine was not quantified so their possible association with adiposity parameters has been not determined.

The effect of cocoa-based product intake on body fat and obesity could be explained by an associated reduction in plasma adipokine (leptin and adiponectin) concentrations, although the mechanisms involved still need to be clarified (8, 47). Leptin and adiponectin are hormones mainly secreted by adipose tissue and delivered into the systemic circulation to modify glucose and lipid metabolism, insulin sensitivity, and cardiovascular function (48). Leptin promotes fatty acid oxidation and reduces lipogenesis by regulating peripheral metabolic pathways in skeletal muscle, adipose tissue, the liver, and the pancreas (49). Meanwhile, plasma adiponectin improves insulin sensitivity, activates muscle utilization of glucose, induces muscle and hepatic fatty acid oxidation, and reduces hepatic glucose production (48). In the present study, adipokine levels were not analyzed and their possible relationship with the intake of flavonoids from cocoa-based products was not determined.

The relationship between the consumption of cocoa-based products and adiposity parameters has been attributed to their flavanol (flavan-3-ol) content. Flavanols from cocoa include mainly monomers and polymers of catechin and epicatechin (7, 9, 10, 43). In our exploratory analysis, we observed a negative correlation between catechins, epicatechins, proanthocyanidins (polymers of flavanols), and WHtR. Catechins and epicatechins, both flavanols monomers, are rapidly absorbed from the upper portion of the small intestine and could influence metabolic pathways related to body weight (50, 51). Gutiérrez-Salmeán et al. suggested that epicatechin decreases the expression of proteins associated with mitochondrial function and increases the expression of protein-induced thermogenesis (51). Instead, although proanthocyanidins are the most abundant (poly)phenols in cocoa-based products, they are poorly absorbed in the small intestine due to their large number of hydrophilic hydroxyl groups (9, 50). Most proanthocyanidins reach the colon and are transformed by the gut microbiota into phenylvalerolactones and phenolic acids, such as hydroxyphenylpropionic acid, hydroxyphenylacetic acid, and benzoic acid (43, 50, 52, 53). These microbial metabolites might be responsible in part for health beneficial effects of proanthocyanidins and could be implicated in adipogenesis and lipogenesis mechanisms (6). Results from a cross-sectional study, based on 2,734 women twins aged 18–83 years, revealed that women with a higher dietary intake of proanthocyanidins-rich foods, which included apples and cocoa drinks, had lower fat mass and central fat mass, both measured by dual-energy-X-ray-absorptiometry (54). In another way, according to the results shown by Lee et al., 5-(3',4'-Dihydroxyphenyl)-γ-valerolactone, a microbial flavanols metabolite, reduces lipid accumulation in 3T3-L1 mature adipocytes regulating free fatty acids metabolism through the suppression of the expression of lipogenic proteins (6). However, evidence for the effect of flavanols from cocoa-based products and their microbial metabolites on adipogenesis and lipogenesis metabolic pathways is yet inconclusive and further studies are needed to better understand the mechanisms of action implicated in weight maintenance.

Although cocoa-based products are an important source of flavonoids that might contribute to the improvement of adiposity parameters, their consumption should be promoted with caution, considering that most commercial formulations are high in calories, sugars, and fats (7). Thus, from a public health perspective, cocoa-based products low in fats and sugars might be recommended.

### Limitations and Strengths

A limitation of the present study is its cross-sectional design, which precludes causal assumptions about flavonoid intake from cocoa-based products and differences in adiposity parameters. In addition, data derived from food frequency questionnaires are prone to bias because misreporting is common in dietary self-assessment in adolescents (55). Misreporting in adolescents is associated with several factors, specifically weight status, weight loss or weight maintenance, body image dissatisfaction, and skipping breakfast (56, 57). Adolescents with high values of BMI tend to report a lower consumption of food rich in energy, fats, and sugars, like cocoa-based products. Misreporting may reflect socially desirable answers where adolescents with self-image dissatisfaction are more likely to under-report the consumption of high fat/high sugar foods. Another plausible reason could be that under-eating is the result of a dietary regimen to lose or maintain weight, so there could be a control in the intake of cocoa-based products. Instead, adolescents with normal weight status could real over-eating to reflect higher intakes due to a growth spurt. Regarding the dietary flavonoids assessment, although our validated food frequency questionnaire specifies portion size, measurement error will be present with any assessment of the flavonoid content of cocoa-based commercial products because they depend on the manufacturing process like alkalinization treatment (58). Furthermore, a limitation of using a food frequency questionnaire is that there is no possible way to determine the exact content of flavonoids from specific cocoa-based products since the percentage of cacao varies for each commercial product. In addition to this, flavonoid intake was estimated through a database, which may not reflect the true concentration of compounds reaching the target organs after digestion, absorption, and metabolism. Therefore, the association between flavonoids from cocoa-based products and adiposity parameters might be distorted by the dietary data bias, so these results should be interpreted with caution. Further longitudinal analyses will be necessary to clarify the true direction of these associations.

Strengths of the present study include the large sample size (*n* = 944) of well-characterized participants, the standardization of measures performed in the SI! Program for Secondary Schools trial, and the inclusion of a range of anthropometric variables, not only BMI, to evaluate adiposity.

In conclusion, a higher intake of flavonoids from cocoa-based products was associated with lower adiposity parameters and less probability of abdominal obesity. These findings are relevant for hypothesis generation regarding mechanisms underlying potential therapeutic effects of cocoa flavonoids against obesity and should stimulate further prospective studies and clinical trials to determine the health beneficial effects of cocoa flavonoids on adolescents.

## Data Availability Statement

The datasets presented in this article are not readily available because there are restrictions on the availability of the data for the SI! Program study, due to signed consent agreements around data sharing, which only allow access to external researcher for studies following project purposes. Requestor wishing to access the database used in this study can make a request to the Steering Committee (SC) chair. For the present study, the database was requested from the SC on 24 February 2022. Requests to access the datasets should be directed to gsantos@fundacionshe.org, rodrigo.fernandez@cnic.es, juanmiguel.fernandez@cnic.es, restruch@clinic.cat, lamuela@ub.edu, bibanez@cnic.es, and vfuster@cnic.es.

## Ethics Statement

The studies involving human participants were reviewed and approved by the Ethics Committee of Instituto de Salud Carlos III in Madrid (CEI PI 35_2016), the Fundació Unió Catalana d'Hospitals (CEI 16/41), and the University of Barcelona (IRB00003099). Written informed consent to participate in this study was provided by the participants' legal guardian/next of kin.

## Author Contributions

RL-R: conceptualization. EL-S, AT-R, and RL-R: methodology. EL-S and CA-R: formal analysis. EL-S, CA-R, and RL-R: investigation. AC-G: data curation. EL-S, CA-R, AT-R, and RL-R: writing—original draft preparation. AT-R, RF-J, JF-A, GS-B, MM, PB, AC-G, CR, AR-L, SC-B, RC, RE, and RL-R: writing—review and editing. EL-S: visualization. AT-R and RL-R: supervision. RF-J, JF-A, GS-B, MM, PB, AC-G, JM-G, AT-R, RE, and RL-R: funding acquisition. All authors have read and agreed to the published version of the manuscript.

## Funding

The SI! Program for Secondary Schools trial was supported by the SHE Foundation, the la Caixa Foundation (LCF/PR/CE16/10700001), the Fundació la Marató de TV3 (grant number 369/C/2016), and by the funding from Idilia Foods (FBG 311240). Support was also provided by the Ministerio de Ciencia, Innovación y Universidades (PID2020-114022RB-I00), CIBEROBN from the Instituto de Salud Carlos III, ISCIII from the Ministerio de Ciencia, Innovación y Universidades (AEI/FEDER, UE), and Generalitat de Catalunya. JM-G was a postgraduate fellow of the Ministerio de Ciencia e Innovación of Spain at the Residencia de Estudiantes (2020–ongoing). RF-J was a recipient of grant PI19/01704 funded by the Fondo de Investigación Sanitaria- Instituto de Salud Carlos III (ISCIII) and co-funded by the European Regional Development Fund/European Social Fund a way to make Europe/Investing in your future. The CNIC was supported by the ISCIII, the Ministerio de Ciencia e Innovación (MCIN), the Pro CNIC Foundation, and was a Severo Ochoa Center of Excellence (CEX2020-001041-S). GS-B was the recipient of grant LCF/PR/MS19/12220001 funded by la Caixa Foundation (ID 100010434). AT-R is a Serra Húnter fellow. EL-S was a FI-SDUR (EMC/3345/2020) fellowship from the Generalitat de Catalunya.

## Conflict of Interest

RL-R reports receiving lecture fees from Cerveceros de España and receiving lecture fees and travel support from Adventia and Idilia Foods SL. RE reports grants from Fundación Dieta Mediterránea, Spain, Cerveza y Salud, Spain, personal fees for given lectures from Brewers of Europe, Belgium, Fundación Cerveza y Salud, Spain, Pernaud-Ricard, Mexico, Instituto Cervantes, Alburquerque, USA, Instituto Cervantes, Milan, Italy, Instituto Cervantes, Tokyo, Japan, Lilly Laboratories, Spain, Wine and Culinary International Forum, Spain, non-financial support to organize a National Congress on Nutrition, and also feeding trials with products from Grand Fountain and Uriach Laboratories, Spain. The remaining authors declare that the research was conducted in the absence of any commercial or financial relationships that could be construed as a potential conflict of interest.

## Publisher's Note

All claims expressed in this article are solely those of the authors and do not necessarily represent those of their affiliated organizations, or those of the publisher, the editors and the reviewers. Any product that may be evaluated in this article, or claim that may be made by its manufacturer, is not guaranteed or endorsed by the publisher.

## Abbreviations

BMI: body mass index
FDR: false discovery rate
FM: fat mass
FMI: fat mass index
SI: *Salud Integral*
WC: waist circumference
WHtR: waist-to-height ratio.
